# Ultrafast interphase exciton funneling in alternating-cation 2D perovskites through Förster-type energy transfer

**DOI:** 10.1039/d6sc06761a

**Published:** 2026-10-13

**Authors:** Supriya Ghosh, Peiran Wang, Heinz Amenitsch, Sumea Klokic, Pavel Chabera, Christian Sternemann, Bapi Pradhan, Johan Hofkens, Tönu Pullerits

**Affiliations:** a Division of Chemical Physics and NanoLund, Lund University Box 124 22100 Lund Sweden tonu.pullerits@chem.lu.se supriya.ghosh@kuleuven.be +46 46 222 81 31 +32 466 34 44 34; b Department of Chemistry, KU Leuven Celestijnenlaan 200F 3001 Heverlee Belgium bapi.pradhan@kuleuven.be +32 465 43 23 04; c Max Planck Institute for Polymer Research Ackermannweg 10 55128 Mainz Germany johan.hofkens@kuleuven.be +32 16 32 78 04; d Institute of Inorganic Chemistry, Graz University of Technology Stremayrgasse 9/IV Graz 8010 Austria; e Department of Physics, College of Science, United Arab Emirates University P.O. Box 15551 Al Ain United Arab Emirates; f Fakultät Physik/DELTA, Technische Universität Dortmund 44221 Dortmund Germany

## Abstract

Efficient exciton transport is central to the development of low-dimensional semiconductors for optoelectronic applications. Layered metal halide perovskites provide a versatile platform for regulating excitonic interactions through spacer-layer engineering. Here, we demonstrate that alternating-cation-in-the-interlayer space (ACI) perovskites exhibit markedly rapid interphase exciton funneling compared with conventional Ruddlesden–Popper (RP) and Dion–Jacobson (DJ) architectures. Using femtosecond transient absorption and time-resolved photoluminescence spectroscopy, complemented by full-spectrum global analysis, we reveal rapid exciton transfer from lower-*n* toward higher-*n* quantum wells. The concomitant decay of lower-*n* excitonic signals and growth of higher-*n* features, together with the species-associated spectral evolution, are consistent with a predominantly Förster-type interphase energy-transfer mechanism. The characteristic early-time dynamics correlate with shorter interlayer spacing along the stacking direction, suggesting that spacer-layer architecture contributes to modulation of interphase excitonic coupling. While differences in phase distribution, spectral overlap, and domain morphology may additionally influence the measured dynamics, these results establish spacer-layer engineering as an effective strategy for regulating ultrafast energy flow in layered excitonic semiconductors.

## Abstract 2

Ultrafast exciton funneling in ACI 2D perovskites occurs through Förster-type energy transfer, enabling efficient energy migration between phases of different thicknesses.

## Graphical abstract

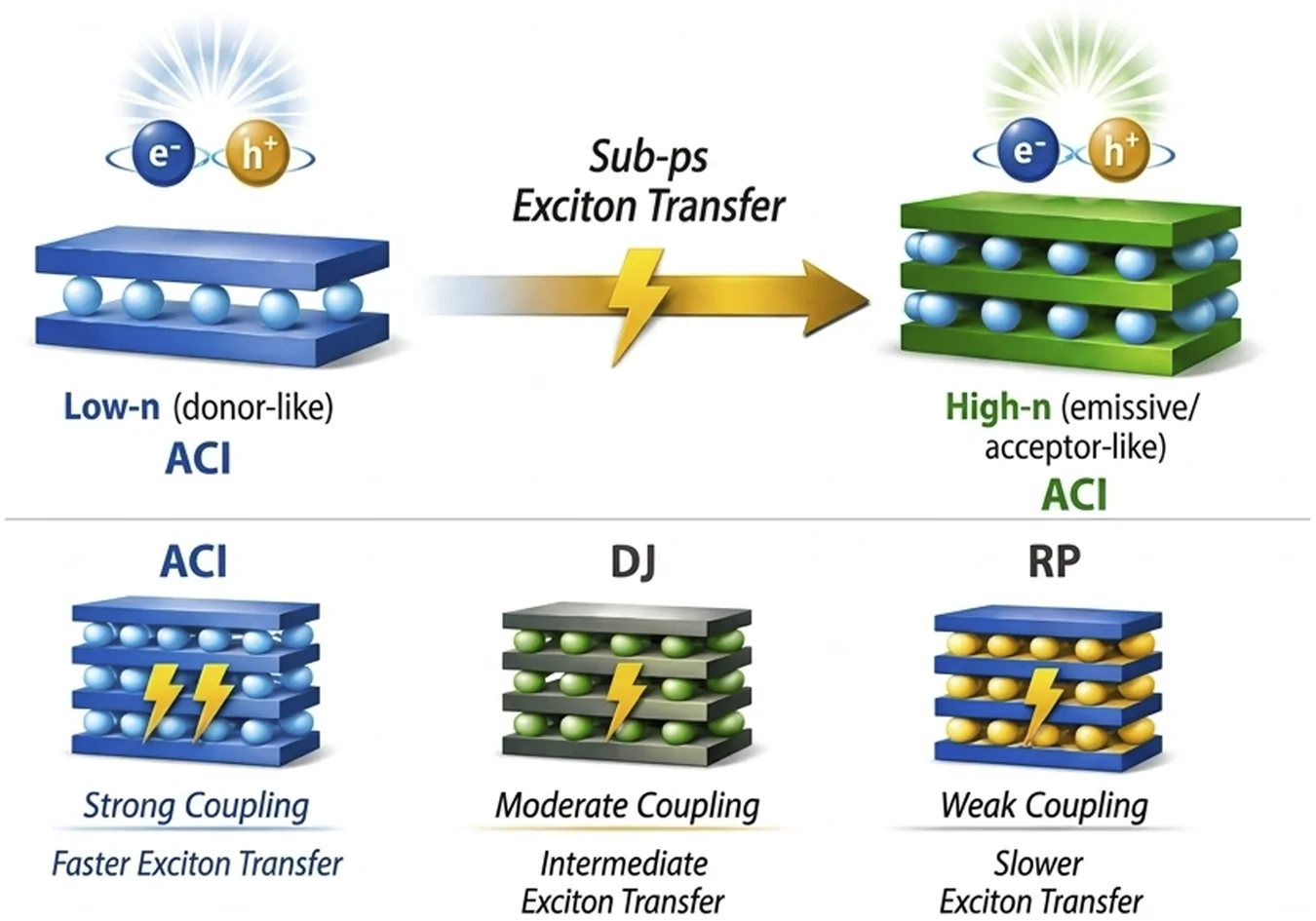

## Introduction

Two-dimensional (2D) perovskites provide an effective route to improving stability by introducing organic spacer cations that separate inorganic lead-halide slabs.^1–4^ This layered architecture enhances resistance to moisture and thermal stress while preserving favorable optoelectronic properties.^5–7^ Among the various 2D perovskite families, Ruddlesden–Popper (RP) perovskites have been most extensively studied,^8–10^ followed by Dion–Jacobson (DJ) structures, which employ divalent spacer cations to reduce the separation between adjacent inorganic layers and facilitate charge transport.^11–13^

More recently, alternating-cation-in-the-interlayer space (ACI) perovskites have been introduced as a distinct class of layered perovskites, featuring two different organic cations arranged alternately within the interlayer region.^14^ This ordered cation configuration leads to reduced interlayer spacing, enhanced structural rigidity, and modified electronic coupling between adjacent inorganic layers.^14^ As a result, ACI perovskites have demonstrated promising performance in photovoltaic and light-harvesting applications, motivating deeper investigation of their excited-state dynamics. From an application standpoint, ACI-based photovoltaics have achieved power conversion efficiencies of around 19% and an open-circuit voltage of approximately 1 V for ACI based photovoltaics.^14–17^

The unique nature of 2D perovskites is the presence of multiple quantum wells (phases with different *n* values), which provide a natural platform for interphase energy and charge transfer.^18–21^ In light-emitting diodes (LEDs), such transfer processes funnel excitons into lower-bandgap emissive domains before nonradiative recombination occurs, thereby improving radiative efficiency and device brightness.^22–24^ In perovskite lasers, carrier transfer along the quantum wells concentrates population in large-*n* phases, facilitating population inversion and reducing lasing thresholds.^25^ For solar cells, band alignment between low-*n* and high-*n* phases promotes electron transfer toward high-*n* phase and hole transfer in the opposite direction, enabling internal charge separation across the film thickness.^26^ This interphase transport not only supports long-range carrier separation but also suppresses recombination losses.^27–29^ Collectively, these studies highlight the critical role of energy and charge transfer in determining the device-relevant photophysics of 2D perovskites.

Previous studies have established ultrafast exciton funneling and Förster-type interphase energy transfer as key photophysical processes in mixed-dimensional Ruddlesden–Popper (RP) perovskites using transient absorption and time-resolved photoluminescence spectroscopy. Panuganti *et al.*^30^ developed a quantum-well model describing exciton energy transfer as a function of donor–acceptor separation, while Proppe *et al.*^31^ demonstrated sub-picosecond exciton funneling in mixed-phase RP perovskites through combined ultrafast spectroscopy and theoretical modeling. More recently, Zhang *et al.*^32^ observed rapid energy transfer between quasi-2D perovskite layers on a timescale of 100–200 fs and examined its influence on exciton-phonon coupling. Williams and co-workers further elucidated the mechanisms governing energy transfer in layered perovskites,^33^ and subsequent studies by Zheng *et al.*^34^ expanded the understanding of exciton funneling in mixed-dimensional perovskite systems. More recently, Xue Lou *et al.*^21^ directly observed robust out-of-plane exciton transport governed by sub-picosecond Förster energy transfer in layered RP perovskites, demonstrating that interlayer exciton transfer can remain remarkably efficient despite the presence of insulating organic spacer layers. Similarly, Saykar *et al.*^35^ reported sub-picosecond interphase energy transfer in quasi-two-dimensional Dion–Jacobson (DJ) perovskites, demonstrating that efficient exciton funneling is also a characteristic feature of DJ structures.

Despite these significant advances, analogous investigations of ACI perovskites remain largely unexplored. More importantly, a systematic experimental comparison of exciton-transfer dynamics among representative ACI, RP, and DJ perovskites investigated under comparable spectroscopic conditions has not yet been reported. Such a comparison is particularly important because these materials possess distinct inorganic layer stacking, organic cation arrangements, interlayer separations, dielectric confinement, and electronic coupling, all of which are expected to influence donor–acceptor coupling and interphase exciton transfer. Consequently, it remains unclear whether the unique alternating-cation arrangement in ACI perovskites influences ultrafast exciton funneling compared with the more extensively studied RP and DJ architectures.

In this work, we perform a systematic ultrafast spectroscopic comparison of representative mixed-phase ACI, RP, and DJ perovskites. While previous work has established the distance dependence of Förster-type energy transfer through systematic variation of spacer-chain length, the present study addresses how fundamentally different spacer and stacking architectures influence interphase exciton dynamics. By combining broadband transient absorption spectroscopy, full-spectrum global analysis, structural characterization, and electromagnetic modeling of interwell energy transfer, we reveal pronounced differences in interphase exciton-funneling dynamics among the ACI, RP, and DJ films. These results extend the established distance-dependent picture by highlighting that comparisons of interphase energy flow across distinct 2D perovskite architectures must account not only for structural separation but also for differences in phase organization, dielectric environment, interlayer structural connectivity, structural rigidity, and excitonic coupling.

## Results and discussion

To investigate interphase exciton dynamics in ACI perovskites, we prepared ACI1, ACI2, and mixed-phase ACI3 films with dominant optical contributions from the *n* = 1 and *n* = 2 excitonic states.^14,36^ Several thin-film deposition routes were evaluated, and the final fabrication procedure was optimized by modifying previously reported hot-casting methods, in which the substrates are heated before spin-coating to promote uniform film formation and preferred orientation.^14^ In this approach, a relatively higher proportion of the *n* = 1 component was intentionally employed to suppress the formation of higher-*n* phases by restricting the availability of guanidinium (GA) and methylammonium (MA) necessary for the growth of thicker perovskite quantum wells. Consequently, increasing the *n* = 1: *n* = 2 ratio results in a donor-rich environment (the *n* = 1 phase represents the exciton donor, and the *n* = 2 phase serves as the dominant acceptor). Importantly, films comprising mixed phases (ACI3) retain well-resolved spectral signatures corresponding to both donor and acceptor components. Moreover, the absorption of the mixed-phase films shows minimal contribution from additional phases, such as higher-*n* quantum wells (Fig. S1a).

To investigate the crystallographic structure and preferred orientation of the ACI (*n* = 1–3) perovskite thin films, grazing-incidence wide-angle X-ray scattering (GIWAXS) measurements were performed. The corresponding two-dimensional GIWAXS patterns, shown in Fig. 1a–c, display diffraction rings accompanied by distinct Bragg spots, indicating the presence of polycrystalline films with some degree of preferential orientation. Three-dimensional structural indexing was carried out to determine the orientation of the crystalline domains relative to the substrate, following previously established protocols.^36,37^ In the out-of-plane scattering direction, pronounced reflections are observed at *q*_z_ = 0.69 Å^−1^ for ACI1 and at *q*_z_ = 0.85 Å^−1^ for both ACI2 and ACI3 (Fig. 1d–f). These peaks are assigned to the (002), (004), and (006) diffraction planes of ACI1, ACI2 and ACI3, respectively, which are characteristic of layered two-dimensional perovskite structures and confirm the preferential alignment of the perovskite lattices with the crystallographic *c*-axis oriented perpendicular to the substrate surface. This preferential orientation is consistently observed across all compositions. GIWAXS results further reveal that ACI1 contains diffraction features associated mainly with the *n* = 1 phase, whereas ACI2 exhibits a phase pure *n* = 2 structure (Fig. 1d and e). In contrast, the ACI3 film shows the coexistence of the phases *n* = 1, 2 and 3, as evidenced by reflections that can be uniquely assigned to individual phases alongside reflections common to multiple phases, indicating phase mixtures (Fig. 1f). This coexistence is accompanied by a markedly higher degree of structural ordering. This is evidenced by the significantly increased diffraction intensity and is consistent with previous reports on ACI-*n* perovskite systems.^38^ The coherence lengths were estimated from the GIWAXS peaks using the Scherrer equation (Table S1).^39^ The values represent lower-limit estimates because instrumental resolution and footprint broadening were not deconvoluted. The ACI1 phases exhibit coherence lengths of 14.3 nm, the ACI2 phase of 11.3 nm, whereas the ACI3 phase exhibited larger coherence lengths of 23.1–25.7 nm. These differences suggest variations in the extent of vertical crystalline ordering among the different ACI phases. Although GIWAXS indicates the coexistence of *n* = 1, *n* = 2 and *n* = 3 phases in ACI3, the TA spectra are dominated by the *n* = 1 and *n* = 2 excitonic resonances, together with an additional weaker bleach feature at longer wavelength. However, because its specific phase assignment cannot be unambiguously established from the available structural and optical data, the quantitative discussion of the early-time interphase transfer focuses on the dominant *n* = 1 and *n* = 2 features. Furthermore, while a minor *n* = 2 contribution in ACI1 cannot be completely excluded based on the GIWAXS measurements, the optical response is predominantly governed by the *n* = 1 excitonic resonance. The surface morphology of the ACI perovskite thin films was examined by scanning electron microscopy (SEM), as shown in Fig. S2. All three compositions exhibit smooth, dense, and homogeneous film coverage over large areas. In the case of ACI3, elongated, rod-like surface features were observed, in agreement with earlier reports on (GA)(MA)PbI_3_ derived perovskite systems.^17^

**Fig. 1:**
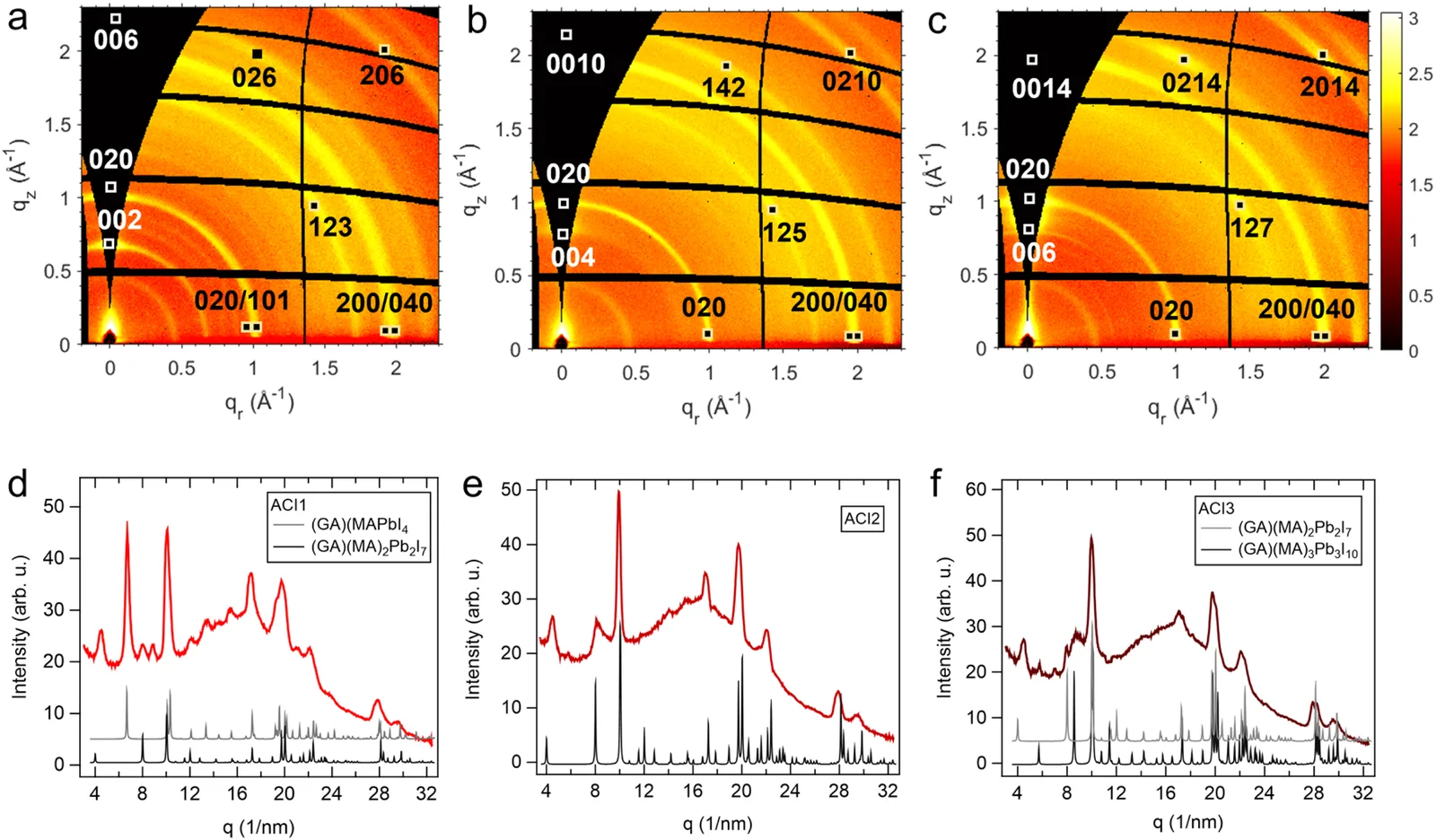
Two-dimensional GIWAXS patterns of (GA)(MA)_*n*_Pb_*n*_I_3*n*+1_ films were recorded at incident angles of 1.3° and above the critical angle (a) ACI1 (b) ACI2 and (c) ACI3. Azimuthal integrations of the GIWAXS patterns are shown alongside simulated patterns derived from the crystal structures of (d) ACI1 (e) ACI2 and (f) ACI3.

Next, interphase exciton energy transfer in the ACI perovskite films was investigated in detail using a transient absorption (TA) spectroscopy. A femtosecond laser pulse at 400 nm excited the samples, and white light continuum was used to probe the photoinduced carrier dynamics.

The TA spectra for three different ACI perovskite films (ACI1, ACI2 and ACI3) are shown in Fig. 2a–c. To minimize contributions from multiexcitonic effects which can obscure the energy-transfer dynamics, all measurements were performed under low-fluence excitation conditions (2.45 µJ cm^−2^ per pulse). Upon above-bandgap excitation, ground-state bleaching (GSB) appears as negative Δ*A* features centered at 583 and 619 nm for ACI1 and ACI2, respectively, while in ACI3 both bleach bands appear (Fig. 2). In addition to the bleach signals, photoinduced absorption features emerge at both lower and higher energies relative to each ground-state bleach. To examine the phase-specific dynamics, the kinetic traces monitored at 583 and 619 nm, corresponding to the *n* = 1 and *n* = 2 excitonic bleach maxima, respectively, are shown in Fig. 3a and b, for both the single-phase and mixed-phase films. These probe wavelengths were selected to minimize contributions from overlapping spectral features. The GSB maxima remain consistent with the corresponding steady-state absorption resonances and exhibit no appreciable spectral shift over the early-time region relevant to the energy-transfer dynamics. Furthermore, the steady-state photoluminescence is Stokes-shifted relative to the donor GSB maximum, minimizing potential stimulated-emission contributions at the selected donor probe wavelength. The kinetics at 583 and 619 nm therefore predominantly track the temporal evolution of the *n* = 1 donor and *n* = 2 acceptor excitonic bleach signals, respectively.

**Fig. 2:**
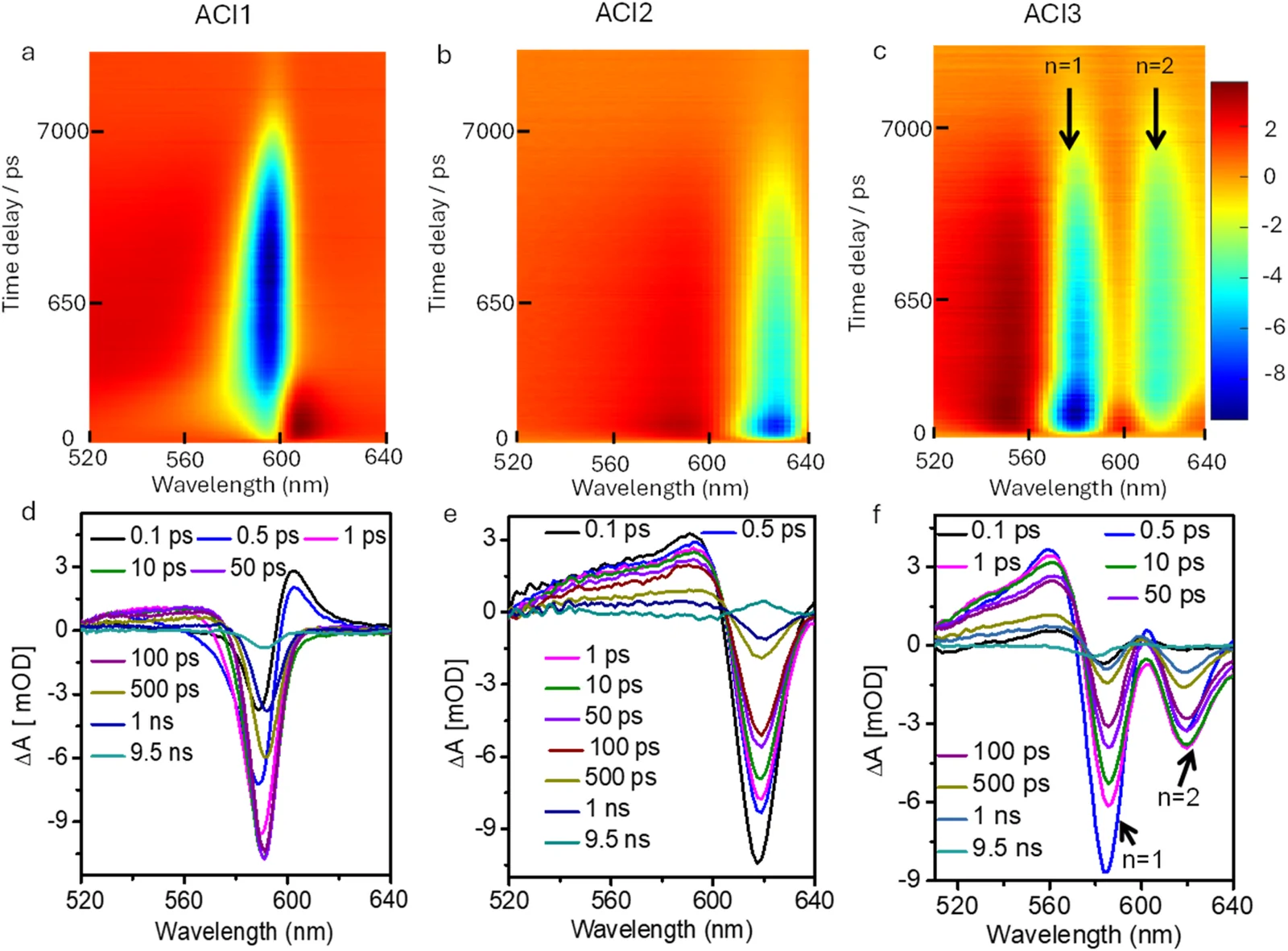
Contour plot of transient absorption (TA) spectra of (a) ACI1, (b) ACI2 and (c) ACI3 perovskite films. Evolution of TA spectra at selected delay times for (d) ACI1, (e) ACI2 and (f) ACI3 perovskite films recorded under 400 nm excitation at a laser fluence of 2.45 µJ cm^−2^, corresponding to the low-excitation regime.

**Fig. 3:**
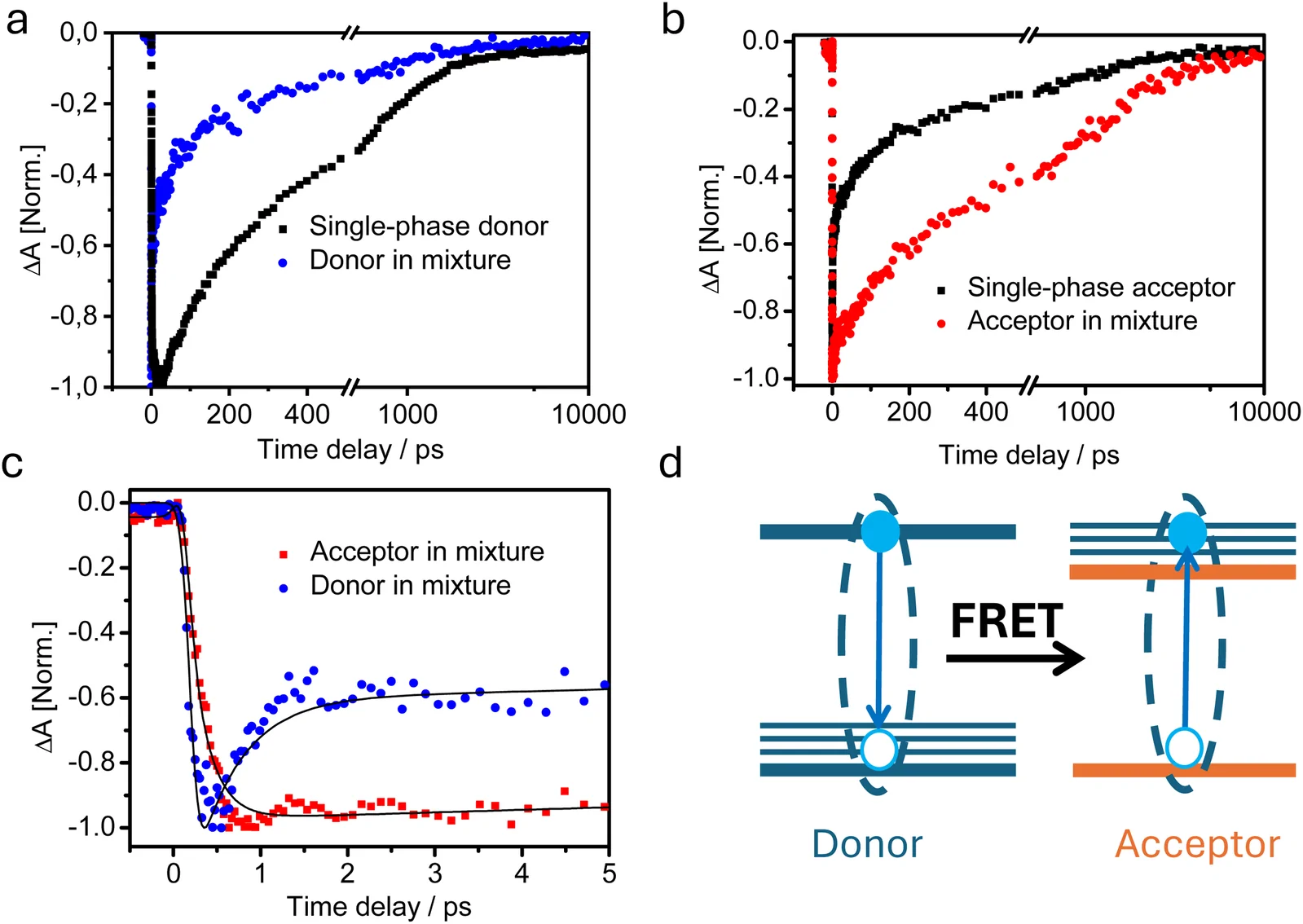
Ultrafast optical response of *n* = 1 and *n* = 2 single and mixed-phase ACI films. (a) Normalized kinetic traces monitored at 583 nm for the donor phase, comparing the single-phase *n* = 1 film with the mixed-composition film. (b) Normalized kinetic traces monitored at 619 nm for the acceptor phase, comparing the single-phase *n* = 2 film with the mixed-composition film. (c) Early-time kinetics of donor and acceptor in mixed-composition film. (d) Schematic representation of resonance energy transfer from donor to acceptor phase.

In the mixed-phase ACI3 film, the donor exciton signal decays more rapidly than in the corresponding ACI1 film (Fig. 3a), while the acceptor signal exhibits a correlated early-time buildup (Fig. 3b). This behavior is consistent with resonant donor-to-acceptor energy transfer.^30^ The temporal correlation between donor depopulation and acceptor growth within the first few picoseconds provides a characteristic spectroscopic signature of interphase energy transfer (Fig. 3c). Interphase dynamics were therefore initially evaluated by comparing the transient responses of the mixed ACI3 film with those of the corresponding ACI1 and ACI2 reference films. The accelerated donor decay and correlated acceptor growth yield an effective sub-picosecond interphase transfer timescale of approximately 0.35 ps.

To further evaluate this transfer timescale, the donor dynamics of the single-phase ACI1 film and the corresponding donor response in ACI3 were compared. The kinetics were normalized at longer delay times, where the dynamics are predominantly governed by slower excited-state relaxation, allowing the additional early-time donor depopulation in the mixed-phase film to be isolated. The difference between the normalized traces is well described by a single-exponential decay with a characteristic time comparable to that obtained from the donor-decay/acceptor-growth analysis (Fig. S3). Because the acceptor is also directly excited at 400 nm, its early-time response contains a prompt, pulse-limited contribution in addition to the delayed growth associated with donor-to-acceptor transfer. Thus, the correlated acceleration of the donor decay and delayed growth of the acceptor bleach provide the primary wavelength-resolved evidence for rapid interphase energy funneling in ACI3. Because excitation at 400 nm initially generates a non-equilibrium exciton/carrier population, relaxation and cooling processes may overlap with interphase energy transfer during the first few hundred femtoseconds. The *n* = 1 excitonic bleach develops within approximately 150–200 fs, comparable to the temporal resolution of the experiment (∼120 fs), and therefore the earliest thermalization dynamics cannot be independently resolved. Consequently, the thermalization state of the transferring population cannot be determined unambiguously. Nevertheless, the subsequent decay of the *n* = 1 donor-associated bleach, accompanied by the correlated rise of the *n* = 2 acceptor-associated bleach, supports ultrafast interphase population transfer on a timescale of ∼350 fs. Accordingly, we interpret the ∼350 fs value as a characteristic timescale for the observed interphase population transfer, rather than assigning it exclusively to an isolated microscopic Förster transfer step.

To determine whether similar interphase dynamics occur across different layered perovskite architectures, we extended the optical measurements to mixed-phase Ruddlesden–Popper (RP) and Dion–Jacobson (DJ) perovskites containing different spacer-cation architectures. These materials should not be regarded as perfectly phase-matched analogues of ACI3, but rather as experimentally relevant mixed-phase systems prepared and investigated under comparable optical conditions. Whereas the optical response of ACI3 is dominated by the *n* = 1 and *n* = 2 excitonic resonances, the RP and DJ films exhibit broader multiphase optical responses. GIWAXS measurements of the RP (*n* = 4) 2D perovskite thin film suggest the presence of multiple coexisting phases (Fig. S4). For DJ (*n* = 4) 2D perovskite thin film the diffraction pattern indicates contribution of *n* = 3 while other phases cannot be excluded (Fig. S5). The steady-state absorption and photoluminescence (PL) spectra (Fig. S6a and b) provide insight into exciton relaxation pathways in these systems. The absorption spectra indicate contributions from multiple phases, while the PL spectra show suppressed emission from low-*n* domains accompanied by enhanced emission from lower-energy high-*n* phases, consistent with exciton funneling across quantum wells.

Notably, the overall PL intensity of mixed-phase RP and DJ films is significantly lower than that of ACI (ACI3), indicating increased nonradiative losses or less efficient exciton funnelling in these systems. This trend is further supported by time-resolved PL measurements (Fig. S6c). The ACI film exhibits a comparatively slower decay that is well described by a single-exponential function with a lifetime of ∼20 ns. In contrast, RP and DJ films display biexponential decay behavior, consisting of a fast initial component (830 ps for RP and 580 ps for DJ) followed by a slower decay (11 ns for RP and 23 ns for DJ), resulting in significantly shorter average lifetimes (∼1.2 ns for both systems). This suggests the presence of additional nonradiative relaxation channels in RP and DJ perovskites.^40^

To gain further insight, we employed ultrafast femtosecond spectroscopy. Fig. 4a–d and present the transient absorption spectra and corresponding kinetics of mixed-phase, RP, and DJ films. The coexistence of these spectrally distinct GSB features confirms the formation of a mixed-phase structure, where the low-*n* wells act as exciton donors and the high-*n* domains serve as energy acceptors.^31,34^ The donor and acceptor dynamics were monitored at 600 and 770 nm for RP, and at 610 and 725 nm for DJ, respectively. These wavelengths were selected at characteristic GSB features exhibiting predominantly lower-*n* donor and higher-*n* acceptor character, respectively, thereby minimizing contributions from overlapping spectral features.

**Fig. 4:**
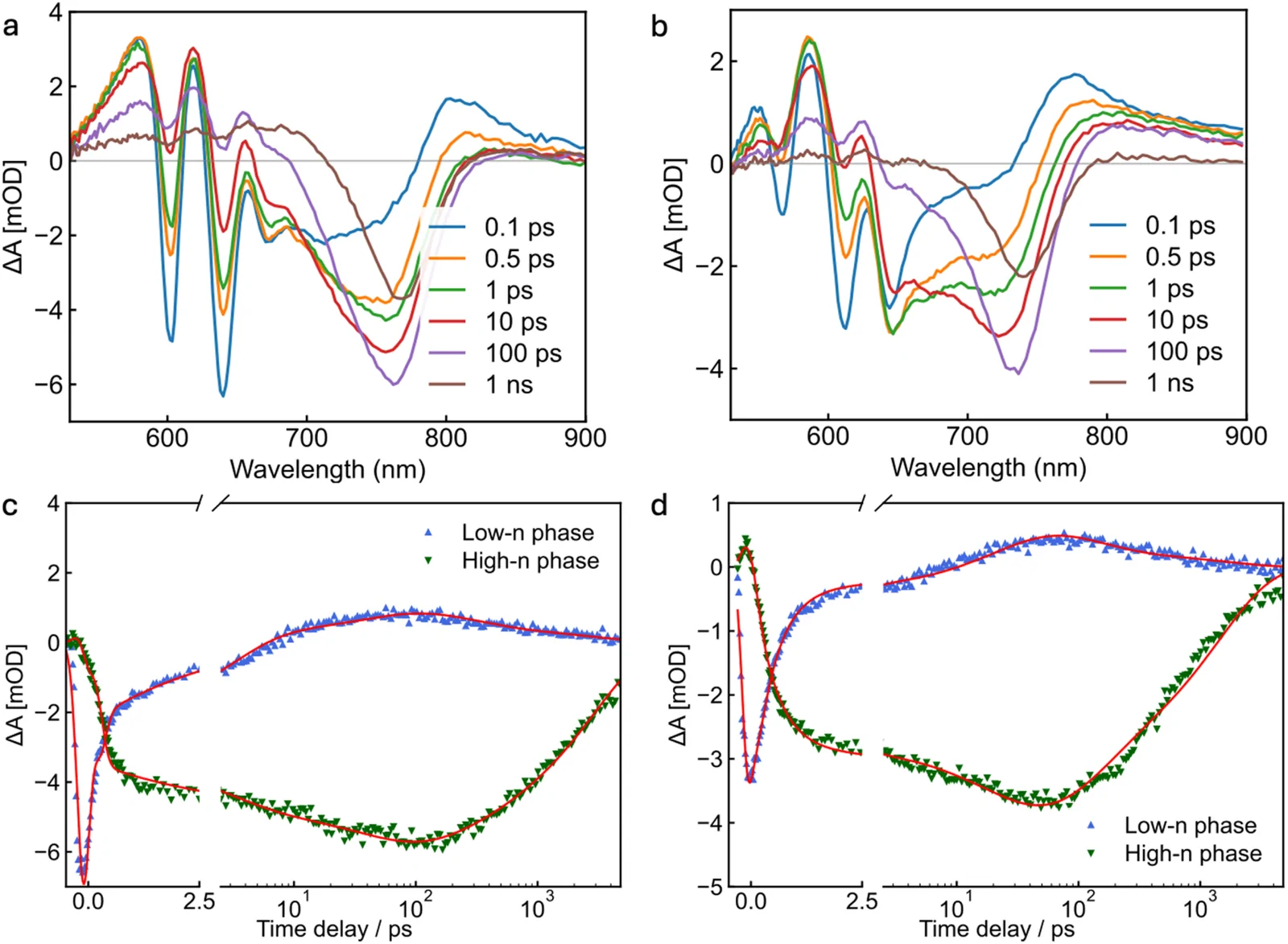
TA spectra of mixed-phase (a) RP perovskite and (b) DJ perovskite. TA kinetics monitored at characteristic wavelengths corresponding to low-*n* (blue) and high-*n* (green) phases for (c) RP and (d) DJ perovskites. The solid red lines are fits to the data.

In both systems, the lower-energy acceptor-associated bleach persists to long delay times, consistent with accumulation and subsequent relaxation of excitation within higher-*n* domains. The early-time kinetics additionally reveal decay of the lower-*n* associated response accompanied by growth of the higher-*n* associated signal, qualitatively consistent with interphase exciton funneling. However, because RP and DJ contain broader and more complex phase distributions than the dominant optical response of ACI3, kinetics extracted at individual wavelengths may contain contributions from multiple overlapping excited-state processes. We therefore avoid assigning the differences among the three architectures solely from independently fitted single-wavelength traces and instead employ full-spectrum global analysis to establish the characteristic kinetic timescales more robustly.

To evaluate the interphase exciton dynamics without relying solely on kinetics extracted at individual probe wavelengths, the TA datasets were subjected to global and target analysis. Singular value decomposition (SVD) was first used to determine the minimum number of significant kinetic components. Three components were sufficient to describe the ACI3 dataset, whereas four components were required for the RP and DJ datasets. The complete global-analysis results, including the concentration profiles, species-associated spectra (SAS), and residual matrices, are presented in Fig. S7.

For ACI3, the global analysis using an instrument response function (IRF, fitted FWHM ≈ 100 fs) convoluted sequential kinetic model yielded characteristic time constants of *τ*_1_ = 0.31 ps, *τ*_2_ = 85.6 ps, and *τ*_3_ = 1.19 ns (Table S2). The SAS exhibit spectral evolution from an early component dominated by the lower-*n* excitonic region toward subsequent components with increased higher-*n* character. Within the sequential kinetic model, the sub-picosecond evolution (*τ*_1_ = 0.31 ps) is consistent with rapid interphase exciton transfer from lower-*n* donor domains toward higher-*n* acceptor domains. This assignment is independently supported by the concomitant decay of the lower-*n* signal and growth of the higher-*n* signal observed in the wavelength-resolved kinetics (Fig. S3c). A weaker long-wavelength bleach feature around 740 nm is also evident in the ACI3 SAS, indicating an additional lower-energy contribution to the transient response. Although this feature may originate from a minor higher-*n* or bulk-like contribution, its specific phase assignment cannot be established unambiguously from the available structural and optical data. We therefore restrict the quantitative phase-specific analysis of the early-time interphase transfer to the dominant *n* = 1 and *n* = 2 excitonic features. Subsequent transfer from the *n* = 2 population toward minor higher-*n* domains cannot be excluded. However, such transfer cannot be quantitatively resolved in the present dataset.

For RP, four characteristic time constants of *τ*_1_ = 2.9 ps, *τ*_2_ = 43.7 ps, *τ*_3_ = 277 ps, and *τ*_4_ = 2.96 ns were obtained (Table S2). The SAS show a progressive redistribution of spectral weight from the lower-*n*-dominated region in the earliest component toward the lower-energy, higher-*n*-dominated region in the subsequent components. This spectral evolution is consistent with the concomitant decay and growth observed in the wavelength-resolved lower-*n* and higher-*n* kinetics (Fig. 4c). Within the sequential model, the initial 2.9 ps component therefore represents a characteristic timescale for interphase exciton funneling toward higher-*n* domains, followed by slower relaxation of the higher-*n* excited-state population. Similarly, for DJ, four characteristic time constants of *τ*_1_ = 0.48 ps, *τ*_2_ = 23.7 ps, *τ*_3_ = 124 ps, and *τ*_4_ = 1.44 ns were obtained (Table S2). The SAS evolve from spectral features predominantly associated with the lower-*n* region toward stronger lower-energy contributions in the subsequent components. This evolution agrees with the complementary lower-*n* decay and higher-*n* growth observed in the wavelength-resolved kinetics (Fig. 4d), supporting rapid interphase exciton funneling. Within the sequential model, the initial 0.48 ps component therefore provides a characteristic timescale for this early interphase evolution, followed by slower relaxation processes extending from tens of picoseconds to the nanosecond regime.

Taken together, the wavelength-resolved kinetics and full-spectrum global analysis provide mutually consistent evidence for exciton funneling from lower-*n* toward higher-*n* domains in all three systems. The characteristic early-time evolution occurs on approximately 0.31 ps for ACI3, 0.48 ps for DJ, and 2.9 ps for RP, revealing substantially faster interphase exciton dynamics in ACI3 and DJ than in RP under the investigated conditions. These characteristic times should not, however, be interpreted solely as intrinsic Förster transfer times, because the measured dynamics may additionally contain contributions from phase distribution, domain morphology, spectral overlap, and exciton migration before reaching donor–acceptor interfaces. We emphasize that the sequential kinetic scheme was imposed as a target model rather than uniquely determined from the TA data. Accordingly, the resulting concentration profiles represent a model-dependent decomposition of the transient spectral evolution, and the early-time constants are interpreted as characteristic interphase energy-funneling timescales rather than intrinsic microscopic FRET rate constants.

The differences in the characteristic early-time dynamics among the three systems correlate with their structural separation along the stacking direction. The corresponding structural spacings along the stacking direction are approximately 0.93 nm for ACI3,^41^ 1.09 nm for DJ,^42^ and 1.56 nm for RP.^43,44^ Notably, the characteristic early-time constants obtained from the global analysis follow the same ordering, increasing from ACI3 (0.31 ps) to DJ (0.48 ps) and RP (2.9 ps). This correlation is qualitatively consistent with stronger dipole-mediated coupling at shorter separations, as expected for a dipole-mediated energy-transfer process. However, these interlayer spacings should not be interpreted as direct microscopic donor–acceptor distances in the polyphase films, and the observed differences in transfer dynamics cannot be attributed to interlayer separation alone. Variations in phase distribution, domain morphology, dielectric environment, spectral overlap, and exciton migration to donor–acceptor interfaces may also contribute to the measured kinetics.^45–47^

The comparatively short interlayer spacing of ACI3 and DJ may favor stronger interphase coupling relative to RP, consistent with their faster early-time exciton-funneling dynamics. In addition, the incorporation of hydrogen-bond-forming cations such as guanidinium in ACI3 and 4-(aminomethyl)piperidinium (AMP) in DJ strengthens the inorganic-organic framework through extensive hydrogen bonding, resulting in a more rigid crystal lattice and enhanced structural stability.^48–54^ These structural characteristics, together with hydrogen-bond-mediated interlayer coupling, may reduce the van der Waals gaps and lower the energetic barriers across the organic layers, thereby facilitating efficient exciton energy funneling in ACI and DJ perovskites from higher-energy, lower-*n* domains toward lower-energy, higher-*n* domains. This rapid interphase transfer can compete effectively with relaxation pathways, such as carrier trapping, nonradiative recombination, and Auger recombination driven by many-body effects, thereby promoting the populations in the lower bandgap emissive domains. This energy-funneling process is particularly relevant for light-emitting diode (LED) application where such transfer processes funnel excitons into lower-bandgap emissive domains before nonradiative recombination occurs, thereby improving radiative efficiency and device brightness as recently established by our group.^36,48^

Taken together, the wavelength-resolved kinetics, full-spectrum global analysis, spectral overlap, and structural characterization are consistent with a dominant Förster-type interphase energy-transfer mechanism. While these observations support a predominantly coulombic, dipole-mediated mechanism, additional short-range contributions arising from wavefunction overlap, exchange interactions, or interwell electronic coupling cannot be excluded, particularly for the more compact ACI and DJ architectures. The present measurements do not allow these contributions to be quantitatively separated. The correlated donor bleach decay and acceptor bleach rise, together with the evolution of the SAS from lower-*n* toward higher-*n* dominated spectral character, provide complementary spectroscopic evidence for rapid energy funneling.

In the weak electronic coupling regime, interwell exciton transfer can be described within the framework of Förster resonance energy transfer (FRET). Similar quantum-well-to-quantum-well energy-transfer models have previously been applied to a variety of low-dimensional semiconductor systems.^30,31^ Within this formalism, the energy-transfer rate is given by1
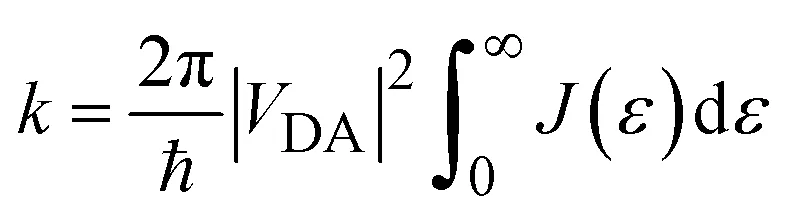
where *V*_DA_ represents the electronic coupling matrix element between donor and acceptor quantum wells, and *J*(*ε*) is the spectral overlap between donor emission and acceptor density of states. Lower-*n* quantum wells exhibit stronger confinement and consequently wider bandgaps, resulting in blue-shifted emission that can overlap efficiently with the higher density of states of higher-*n* quantum wells. This favorable spectral overlap, together with enhanced donor–acceptor coupling, provides basis for interwell exciton transfer.

These considerations are consistent with our TA measurements, which reveal rapid energy funneling from lower-*n* toward higher-*n* excitonic states in all three perovskite architectures. The wavelength-resolved kinetics yield a characteristic interphase population redistribution timescale of approximately 0.35 ps for ACI3, while the full-spectrum global analysis independently identifies a comparable early-time component of 0.31 ps. The corresponding early-time components obtained from the global analysis are 0.48 ps for DJ and 2.9 ps for RP, establishing the trend ACI3 < DJ < RP. The faster early-time dynamics observed for ACI3 and DJ relative to RP are qualitatively consistent with stronger donor–acceptor coupling at shorter structural separations (Fig. 4a–c). However, differences in spectral overlap, phase organization, dielectric environment, and exciton migration may also contribute to this trend. The progressively slower transfer dynamics correlate with the increasing interlayer separation and weaker donor–acceptor coupling in these systems. Because eqn (1) depends on both the electronic coupling and the spectral overlap, it provides a qualitative framework for interpreting the experimentally observed transfer trends among the three perovskite families.

To further examine the distance dependence of the dipole-mediated interaction, we employed the electromagnetic model previously developed for semiconductor nanoplatelet systems. Unlike eqn (1), which provides the general FRET formalism in terms of the electronic coupling *V*_DA_, eqn (2) enables a numerical evaluation of the transfer rate for a specified donor–acceptor geometry. In this approach, the donor quantum well is represented by an effective oscillating transition dipole located at the center of the donor slab, while the transfer rate is determined from the electromagnetic power absorbed within the acceptor slab. The energy-transfer rate is given by^31,55^2
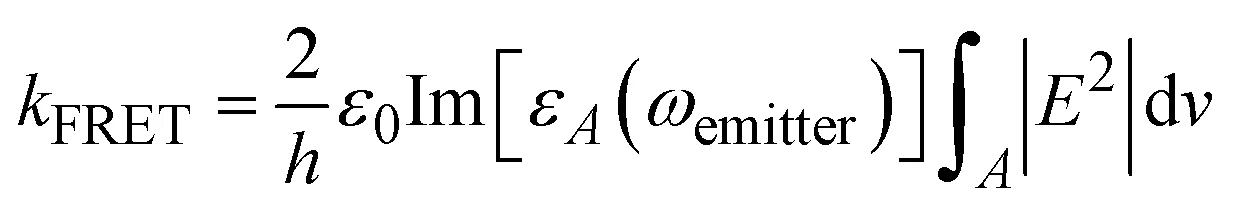
where *ε*_0_ is the vacuum permittivity, *E* is the electric field generated by the donor transition dipole, and *ε*_*A*_ is the complex dielectric function of the acceptor evaluated at the donor emission frequency *ω*_emitter_. The dielectric constant of the organic spacer region is set to 2.38, while the donor and acceptor quantum wells are assumed to have similar dielectric responses.^31^ A donor transition dipole moment of *µ* ≈ 2–4 eÅ (≈10–20 D) has previously been reported for strongly confined 2D lead-halide perovskites.^36,51,56^ For the calculation shown in Fig. 5, a fixed in-plane donor transition dipole moment of *µ* = 2.5 eÅ was used. No model parameter was adjusted or optimized to reproduce the experimentally determined early-time constants. The electric-field intensity generated by the donor dipole was evaluated throughout the acceptor slab, and the volume integral in eqn (2) was calculated numerically to determine the electromagnetic power absorbed within the acceptor. The calculated energy transfer lifetime was subsequently obtained according to *τ* = 1/*k*_FRET_ (Fig. 5).

**Fig. 5:**
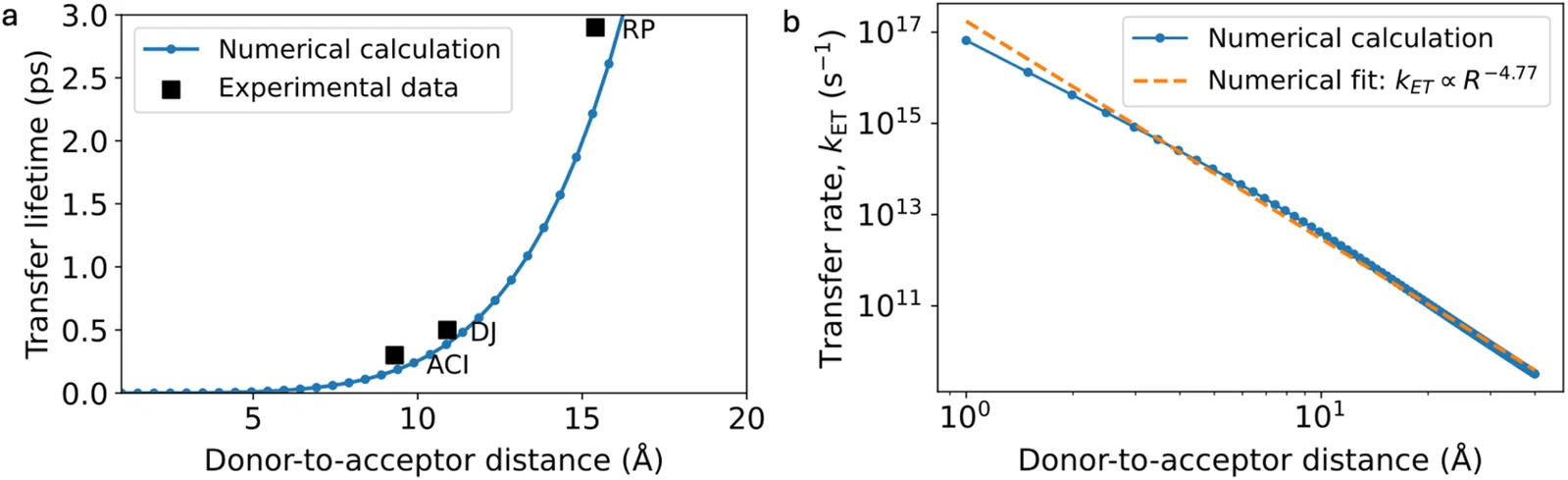
(a) Calculated donor transfer lifetime as a function of donor–acceptor separation based on eqn (2). Black squares indicate the characteristic early-time constants obtained from the global analysis for ACI3, DJ, and RP. (b) Calculated transfer rate as a function of donor–acceptor separation based on eqn (2). The red dashed line represents the fitted power law dependence, *τ* ∝ *R*^4.77^.

Importantly, eqn (2) is distinct from the conventional molecular Förster expression for two point dipoles, for which an *R*^−6^ dependence is obtained in the dipole–dipole limit.^57,58^ Here, although the excitation source is represented by an effective donor transition dipole, the acceptor is treated as a spatially extended slab and the electromagnetic field is integrated over its volume. Consequently, a particular power-law distance dependence is not imposed *a priori* but emerges from the assumed geometry and dielectric response of the model. For the parameters employed here, the calculated transfer rate approximately follows *k*_ET_ ∝ *R*^−4.77^, which corresponds to *τ* ∝ *R*^4.77^.

The calculated distance dependence is qualitatively consistent with the experimentally observed acceleration of the early-time exciton-funneling dynamics with decreasing interlayer spacing. The comparatively short interlayer spacing of ACI3 and DJ may therefore favor stronger interphase coupling relative to RP, consistent with the electromagnetic model and the expected decrease in dipole-mediated coupling with increasing separation. The electromagnetic calculation should, however, be regarded as a mechanistic model rather than a quantitative fit to the three experimental points. The calculated distance dependence is specific to the assumed donor–acceptor geometry, dielectric response, transition dipole moment, and spatial extent of the interacting states. Accordingly, although the experimental trend is consistent with the strong distance dependence predicted by the electromagnetic model, this agreement should not be interpreted as evidence that donor–acceptor separation alone determines the measured transfer lifetime. Differences in phase organization, dielectric environment, transition-dipole orientation, structural rigidity, and exciton migration may also contribute to the observed kinetics, although their individual contributions cannot be quantitatively separated in the present dataset.

The comparison among ACI3, DJ, and RP must additionally account for differences in their underlying phase organization. These films are not structurally or spectroscopically phase-matched analogues and therefore differ not only in interlayer spacing but also in phase distribution, spectral overlap, dielectric environment, and potentially exciton-migration pathways. GIWAXS indicates the coexistence of *n* = 1, *n* = 2, and *n* = 3 phases in ACI3. The TA response is nevertheless dominated by the *n* = 1 and *n* = 2 excitonic resonances, together with a weaker long-wavelength contribution whose specific phase assignment cannot be established unambiguously. Furthermore, the GIWAXS-derived coherence lengths characterize crystalline ordering along the out-of-plane stacking direction and do not directly provide lateral domain dimensions, microscopic donor–acceptor interface densities, or exciton diffusion lengths. Reliable quantitative domain-size estimates could not be obtained for the RP and DJ films from the available diffraction data. Consequently, the ACI3 < DJ < RP ordering of the characteristic early-time dynamics is interpreted as a correlation between interphase energy-funneling dynamics and interlayer spacing in the representative films investigated here, rather than as evidence that donor–acceptor separation or spacer-cation architecture alone determines the transfer rate. Spectral overlap, phase distribution, transition-dipole orientation, dielectric environment, and exciton diffusion may additionally contribute to the measured kinetics.

Thus, rather than identifying donor–acceptor separation as the sole determinant of the transfer rate, our results show that the broader spacer and stacking architecture provides a structural framework for regulating ultrafast interphase energy flow through the combined influence of interlayer separation, phase organization, dielectric environment, structural rigidity, and excitonic coupling.

## Conclusions

In summary, this study demonstrates that spacer-layer architecture strongly influences interphase exciton dynamics in quasi-two-dimensional perovskites. Through a comparative ultrafast spectroscopic investigation of ACI, RP, and DJ perovskites, combined with full-spectrum global analysis, structural characterization, and electromagnetic modeling, we identify rapid energy funneling from lower-*n* toward higher-*n* excitonic states. The characteristic early-time dynamics reveal particularly rapid interphase energy funneling in ACI3 under the investigated conditions. This kinetic trend correlates with the decreasing interlayer spacing from RP to DJ and ACI3 and is qualitatively consistent with enhanced dipole-mediated coupling at shorter interlayer separations. The combined wavelength-resolved kinetics, species-associated spectral evolution, donor–acceptor spectral overlap, and electromagnetic calculations support a predominantly Förster-type mechanism for the observed interphase energy funneling. More broadly, these findings establish spacer-layer engineering as a promising approach for controlling ultrafast energy flow in layered excitonic semiconductors and provide design principles for tailoring exciton transport in optoelectronic materials.

## Author contributions

S. G.: conceptualization, methodology, investigation, formal analysis, software, data curation, visualization, and writing – original draft. P. W.: investigation and methodology. H. A.: investigation, formal analysis, and methodology related to GIWAXS measurements. S. K.: investigation, formal analysis, and methodology related to GIWAXS measurements and structural characterization. P. C.: investigation and methodology. C. S.: investigation, formal analysis, and methodology related to GIWAXS measurements and structural analysis. B. P.: conceptualization, writing, review & editing. J. H.: conceptualization, supervision, resources, funding acquisition, and writing, review & editing. T. P.: conceptualization, supervision, methodology, funding acquisition and writing, review & editing.

## Conflicts of interest

There are no conflicts to declare.

## Supplementary Material

SC-OLF-D6SC06761A-s001

## Data Availability

The data supporting the findings of this study are available within the article and its supporting information. Supplementary information (SI): full experimental details, including the material synthesis, characterization, steady state and time resolved measurements, SEM image, GIWAXS data for RP and DJ perovskite, steady state PL, time resolved PL kinetics, and details of TA kinetic fitting and global analysis. See DOI: https://doi.org/10.1039/d6sc06761a.
