# Supplementary material for: Ultrafast interphase exciton funneling in alternating-cation 2D perovskites through Förster-type energy transfer
Source: Chem Sci. 2026 Oct 13. Online ahead of print. doi: 10.1039/d6sc06761a (PMC13679185; doi:10.1039/d6sc06761a)
Supplement: Supplementary file 1 [file SC-OLF-D6SC06761A-s001.pdf]

Supplementary Information (SI) for Chemical Science.
This journal is © The Royal Society of Chemistry

Supporting Information

Ultrafast Interphase Exciton Funneling in Alternating-Cation 2D Perovskites through Förster-Type Energy Transfer

Supriya Ghosh[†,§*], *Peiran Wang*[§], *Heinz Amenitsch*[⊥], *Sumea Klokic*[⊥], *Pavel Chabera*[†,β], *Christian Sternemann*[ǀ], *Bapi Pradhan*[§*], *Johan Hofkens*[§,δ*], *and Tonu Pullerits*[†,*]

[†]Division of Chemical Physics and NanoLund, Lund University, Box 124, 22100 Lund, Sweden

[§]Department of Chemistry, KU Leuven, Celestijnenlaan 200F, 3001 Heverlee, Belgium

[δ]Max Planck Institute for Polymer Research, Ackermannweg 10, 55128 Mainz, Germany

[⊥]Institute of Inorganic Chemistry, Graz University of Technology, Stremayrgasse 9/IV, Graz 8010, Austria

[β]Department of Physics, College of Science, United Arab Emirates University, P.O. Box 15551, Al Ain, United Arab Emirates

[ǀ]Fakultät Physik/DELTA, Technische Universität Dortmund, 44221 Dortmund, Germany

AUTHOR INFORMATION

Corresponding Authors

Email: tonu.pullerits@chem.lu.se Phone: +46 46 222 81 31

Email: johan.hofkens@kuleuven.be Phone: +32 16 32 78 04

Email: bapi.pradhan@kuleuven.be Phone: +32 465 43 23 04

Email: supriya.ghosh@kuleuven.be Phone: +32 466 34 44 34

Experimental Details

Materials

Methylammonium iodide (MAI, 99%, GreatCell Solar), lead iodide (PbI2, 99.99%, Alfa Aesar), and guanidinium iodide (GAI, 99%, GreatCell Solar) were used as received. Toluene (anhydrous, 99.8%, Merck), dimethyl sulfoxide (DMSO, anhydrous, 99.9%, Sigma-Aldrich), chlorobenzene, diethyl ether and N,N-dimethylformamide (DMF, anhydrous, 99.8%, Sigma-Aldrich) were employed as solvents and antisolvents. All precursor handling and film fabrication steps were carried out inside a nitrogen-filled glovebox.

Preparation of ACI Thin Films

Thin films of alternating-cation-in-interlayer (ACI) perovskites with nominal compositions $(GA)(MA)_nPb_nI_{3n+1}$ (n = 1, 2, and 3) were fabricated using a solution-processing route adapted from previously reported procedures with minor modifications.[1] For each composition (n = 1-3), stoichiometric amounts of MAI, GAI, and PbI_2 were dissolved in a mixed DMF/DMSO solvent system (9:1 by volume), producing precursor solutions with a final concentration of 1.2 M. After that it was allowed to stir magnetically at ambient temperature overnight to achieve full solubilization. Substrates (2.5 × 2.5 cm^2) were subjected to successive ultrasonic cleaning steps in detergent solution, acetone, isopropanol, and ethanol, each for 30 min, followed by drying under nitrogen purge and ozone plasma treatment for 15 min to remove surface contaminants.

All film fabrication steps were conducted under an inert nitrogen environment. The precursor solutions were spin-coated using a two-stage program (500 rpm for 3 s and 4000 rpm for 60 s), during which chlorobenzene (200 μL) was added as an antisolvent near the midpoint of the high-speed step. The films were then thermally treated at 80 °C for 15 min to promote crystallization.

Preparation of Dion-Jacobson (DJ) Perovskite Films

DJ-type perovskite films ($(4AMP)(MA)_3Pb_4I_{13}$, n=4) were prepared from precursor solutions containing PbI_2 (2 mmol), MAI (1.5 mmol), and 4-aminomethylpiperidinium iodide (4amppI2, 0.5 mmol) dissolved in a mixed DMF/DMSO solvent (4:1 by volume), with the addition of hydroiodic acid (16 μL).[2] The solutions were stirred overnight prior to deposition. Cleaned glass substrates were coated inside a nitrogen glovebox using a two-step spin-coating process identical to that described above. During the high-speed spin step, diethyl ether (700 μL) was used as an antisolvent. The deposited films were annealed at 100 °C for 10 min to complete film formation.

Preparation of Ruddlesden-Popper (RP) Perovskite Films

RP-type perovskite ($BA_2MA_3Pb_4I_{13}$, n=4) precursor solutions were prepared by dissolving MAI (1.5 mmol), butylammonium iodide (BAI, 1 mmol), and PbI_2 (2 mmol) in DMF.[3, 4] After overnight stirring, the solution was spin-coated onto glass substrates that had been preheated to 85 °C for 10 min. The glass substrates followed same washing procedure were used for the film preparation. Film deposition was performed in a nitrogen-filled glovebox under inert conditions. The films were deposited at 5000 rpm for 30 s and subsequently annealed at 100 °C for 10 min.

Spectroscopic Techniques

Steady-State Optical Characterization

UV-Vis absorption spectra were recorded using a PerkinElmer Lambda 950 spectrophotometer. Steady-state photoluminescence measurements were carried out using an Edinburgh Instruments FLS980 fluorometer. All optical measurements were performed at room temperature under ambient conditions.

Time-Resolved Photoluminescence

Time-resolved photoluminescence measurements were carried out using a time-correlated single-photon counting (TCSPC) setup. Excitation was provided by the frequency-doubled output of a mode-locked Ti:sapphire laser (Tsunami, Spectra Physics). Fluorescence emission was collected at a right angle (90°) relative to the excitation beam and passed through a polarization filter oriented at the magic angle (54.7°) with respect to the excitation polarization to eliminate anisotropy effects.

The emitted light was spectrally dispersed using a monochromator (Sciencetech 9030; focal length 100 mm; wavelength accuracy 0.3 nm) and detected with a microchannel-plate photomultiplier tube (MCP-PMT, R3809U-51, Hamamatsu). Photon arrival times were recorded using a TCSPC timing module (SPC-830, Becker & Hickl), generating fluorescence decay histograms with 4096-time channels. The instrument response function (IRF) exhibited a typical full width at half maximum (FWHM) of approximately 42 ps.

Fluorescence decay profiles were analyzed by iterative reconvolution of the measured decay with the experimentally determined IRF. The quality of the fits was assessed using multiple statistical criteria, including reduced chi-square values ($\chi^2 < 1$), randomness of residuals ($Z^2 < 3$), and Durbin-Watson parameters within the range 1.8-2.2, together with visual inspection of the residual distributions and their autocorrelation.

Transient Absorption (TA) Measurements

Broadband femtosecond pump-probe measurements were carried out using a Solstice amplified laser system (Spectra-Physics), producing approximately 80 fs pulses centered at 796 nm with a repetition rate of 4 kHz. The laser output was split into two beams for pump and probe generation. Pump pulses were generated using a collinear optical parametric amplifier

(TOPAS-C, Light Conversion). A second TOPAS was used to generate 1350 nm pulses, which were focused into a CaF_2 crystal to produce a broadband white-light continuum probe.[5]

The relative polarization between the pump and probe beams was set to the magic angle, 54.7°, using a Berek compensator placed in the pump beam. The pump and probe beams were spatially and temporally overlapped at the sample position. To reduce laser-induced noise and pulse-to-pulse fluctuations, the probe beam was split into sample and reference beams, which were detected separately. The pump beam was modulated at 2 kHz using a mechanical chopper synchronized to the 4 kHz laser repetition rate, blocking every second pump pulse. The transient absorption spectra were recorded with double diode-array coupled to an in-house-developed prism spectrograph (Pascher Instruments). Correction for group velocity dispersion of the white-light continuum was performed during data processing

Structural Characterization

Scanning Electron Microscope

Surface morphology was investigated by scanning electron microscopy using a JEOL SM-13020RLV instrument operated at 2 kV. Images were recorded at low and high magnifications (×270 and ×3000) to assess large-area uniformity and fine surface features of the films.

Grazing-Incidence Wide-Angle X-Ray Scattering (GIWAXS)

GIWAXS measurements of ACI and RP films have been performed at the Austrian SAXS beamline at the ELETTRA synchrotron in Trieste (Italy) at a photon energy of 8 keV.[6] The beam size was set to 0.1 x 1 mm (V × H) at an adjusted sample to detector (Pilatus3 1M, Dectris) distance of 217.3 mm using silver behenate as a reference pattern. The measurements were performed in air at incident angles of 1.3° to provide depth-sensitive information with the X-ray beam penetrating into the film, with the patterns corrected for the fluctuations of the primary intensity.[7] The GIWAXS patterns were reconstructed and indexed using the GIXSGUI

toolbox.[8] The GIWAXS pattern shown in Figure 1a-c are represented by orthogonal q_z and q_r reciprocal axes, where q_z denotes the wave vector transfer component normal to the surface and q_r being the total in-plane wave vector transfer. In such a representation a missing wedge appears, since the intensity distribution on an area detector measures the projection of the reciprocal lattice being intercepted by the Ewald sphere onto the detector plane. Integration of the GIWAXS pattern to one-dimensional out-of-plane and in-plane pattern was performed by SAXSDog.[7] Simulation of the single-crystal3 patterns was done by Mercury, while intensity profile comparison was performed by IGOR Pro (Wavemetrics).

Table S1. *Estimated domain sizes for ACI 1-3 from the GIWAXS data*

		q (nm^{-1})	FWHM (nm^{-1})	Estimated domain size (nm)
**ACI-1**	n = 1	6.72	0.396 ± 0.006	14.3 ± 0.2
**ACI-2**	n = 2	4.49	0.501 ± 0.009	11.3 ± 0.2
**ACI-3**	n = 1	7.00	0.219 ± 0.022	25.7 ± 2.6
	n = 2	5.76	0.244 ± 0.018	23.1 ± 1.7

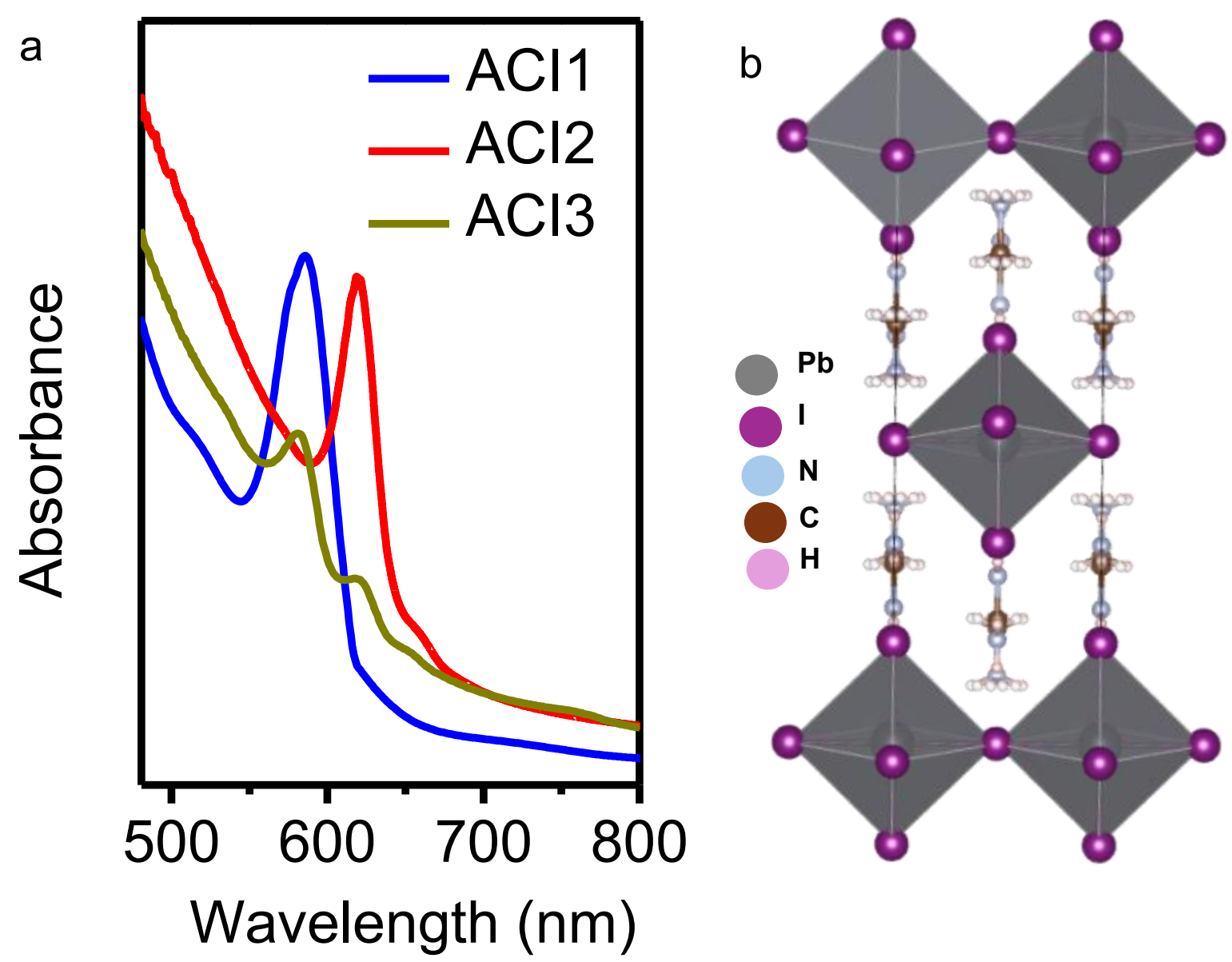

Figure S1. (a) Absorption spectra of ACI1, ACI2 and ACI3 thin films and (b) Crystal structure of ACI3 perovskite.

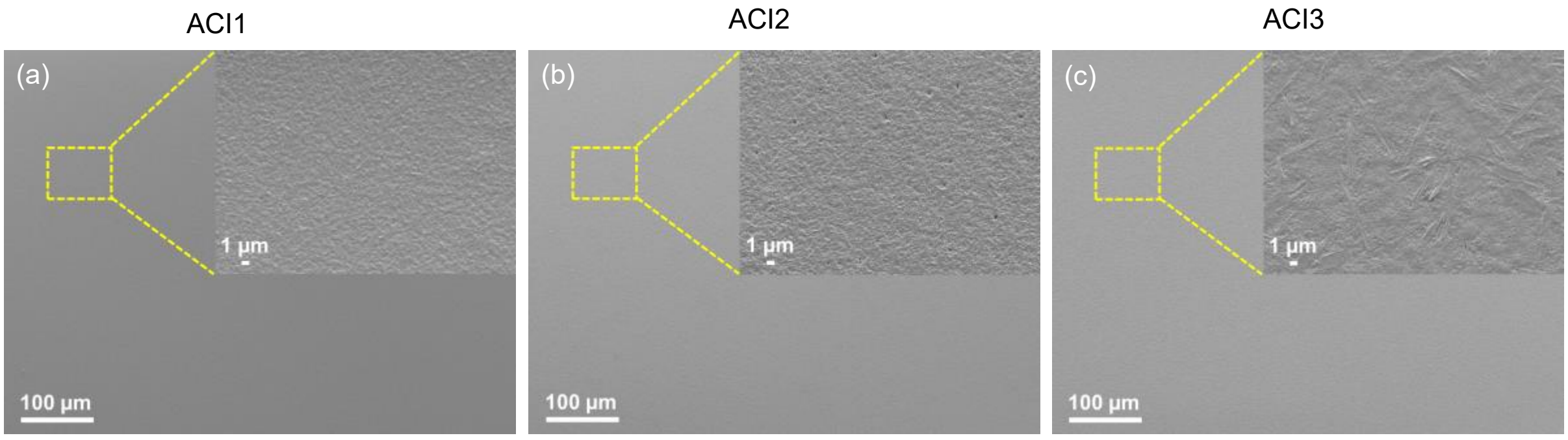

Figure S2. SEM image of (a) ACI1 (b) ACI2 and (c) ACI3 perovskite thin films.

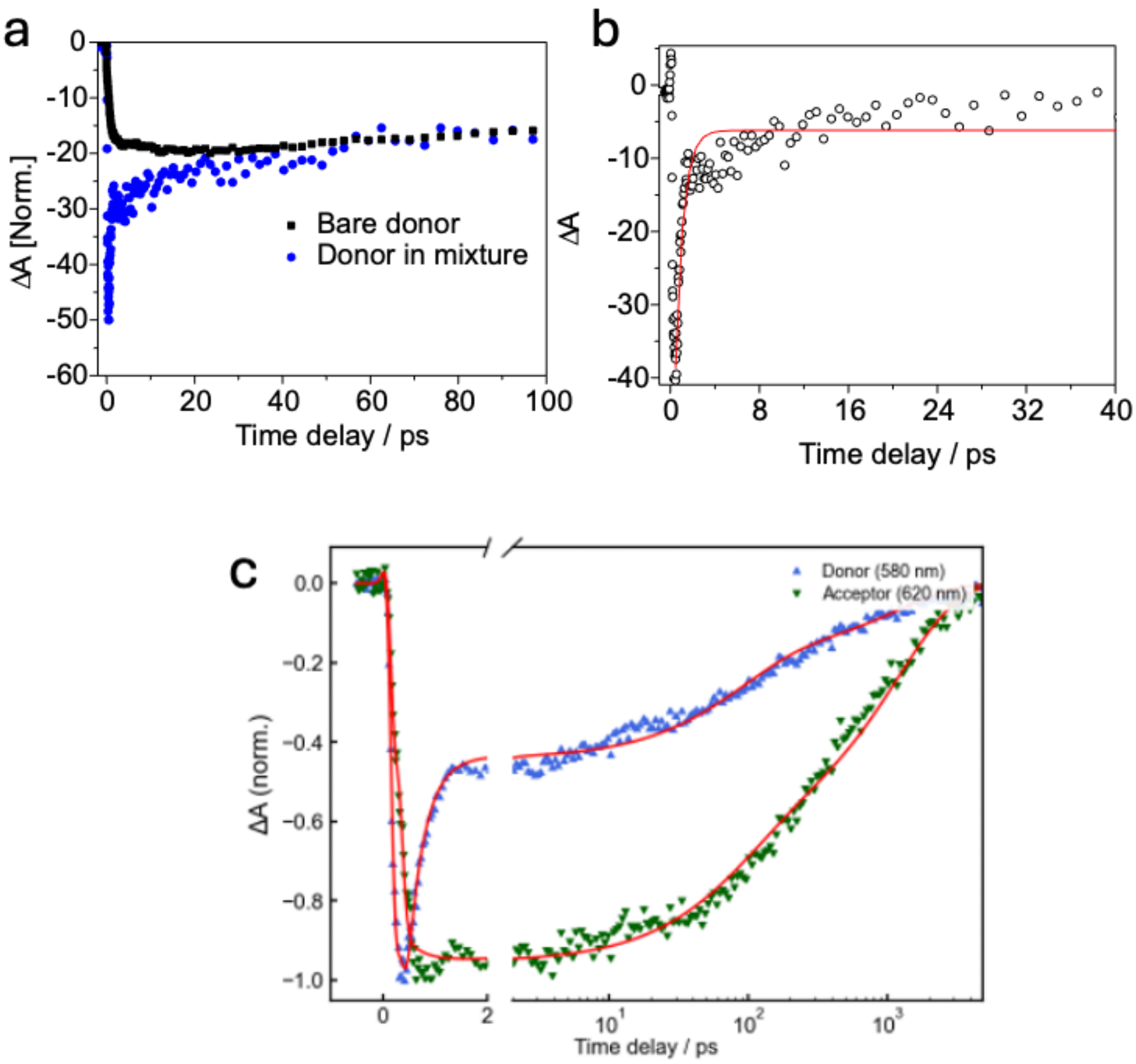

Figure S3. (a) TA dynamics of bare donor (black) and donor in mixture (blue), scaled to the long-lived decay signal. (b) Subtracted TA response between donor in mixture and bare donor with the decay described by a single-exponential fit. (c) Fitted TA dynamics of donor and acceptor in mixed film ACI3. Solid red lines are fits to the data.

Grazing-incidence wide-angle X-ray scattering (GIWAXS) measurements of RP and DJ perovskites

To evaluate the phase composition of the film, grazing-incidence wide-angle X-ray scattering (GIWAXS) measurements were performed at an incidence angle of 1.3°, corresponding to an estimated penetration depth of approximately 440 nm. This incidence angle was deliberately chosen to probe the bulk of the film and thus provide a representative assessment of all phases present. The resulting two-dimensional GIWAXS patterns were azimuthally integrated, as well as along both the out-of-plane and in-plane directions to assess the preferential orientation of the phases with respect to the substrate, as well as any directional growth tendencies. For phase identification, the experimental data were compared with literature and calculated diffraction patterns for $BA_2MA_{n-1}Pb_nI_{3n+1}$ (n = 2, 3, and 4) as well as BA_2PbI_4 (n = 1).[9] This analysis reveals that the film is predominantly composed of the n = 2 phase, with minor contributions from the n = 1 phase, both exhibiting strong out-of-plane reflections. In contrast, the n = 3 and n = 4 phases are present only in small amounts, with the latter appearing primarily in the in-plane direction.

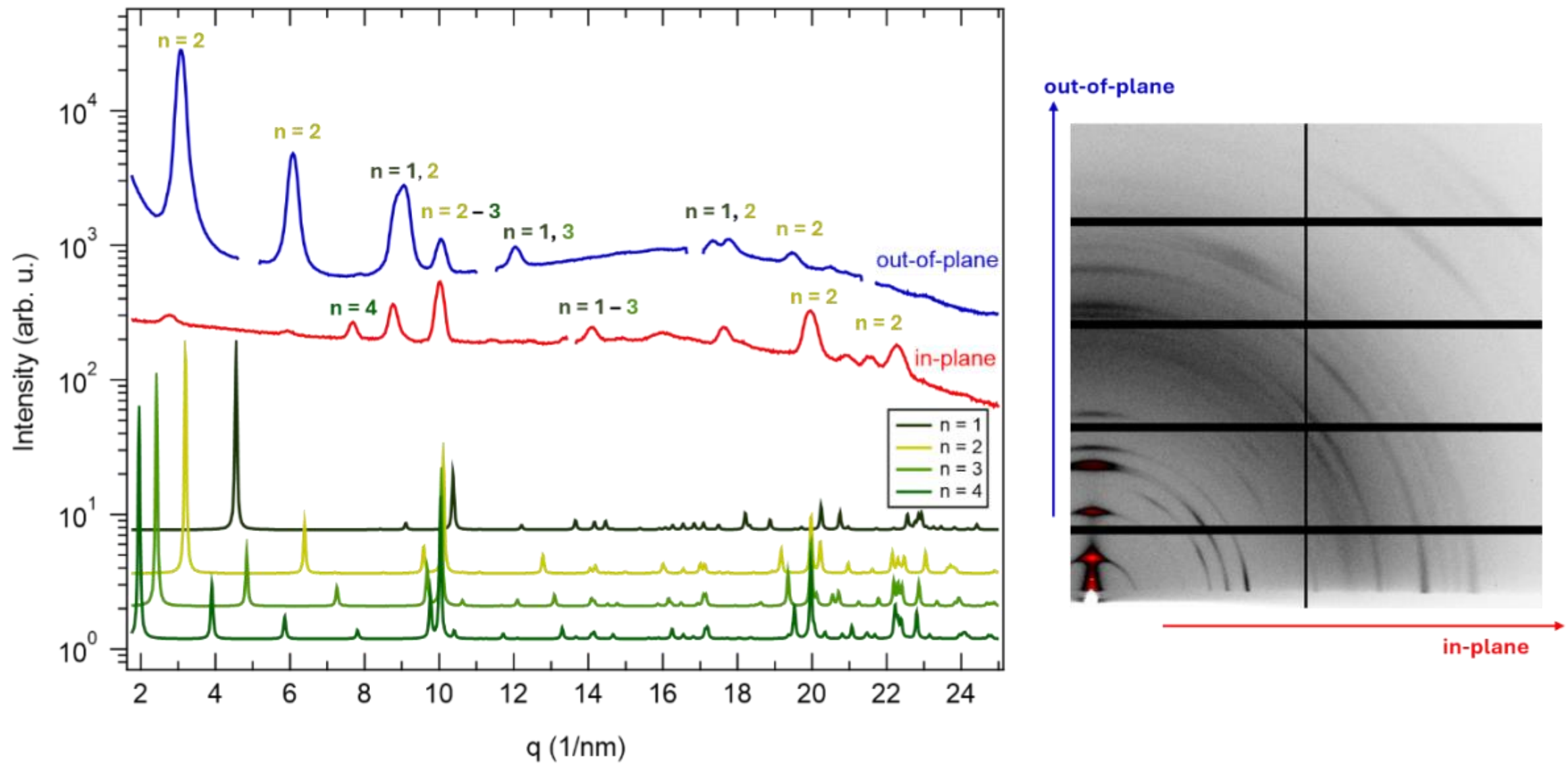

Figure S4. GIWAXS data integrated along the out-of-plane and in-plane directions, with reflections assigned based on calculated diffraction patterns for RP ($BA_2MA_{n-1}Pb_nI_{3n+1}$ (n = 2, 3, and 4)) and BA_2PbI_4 (n = 1). The corresponding 2D GIWAXS pattern, indicating the integration directions, is shown on the left.

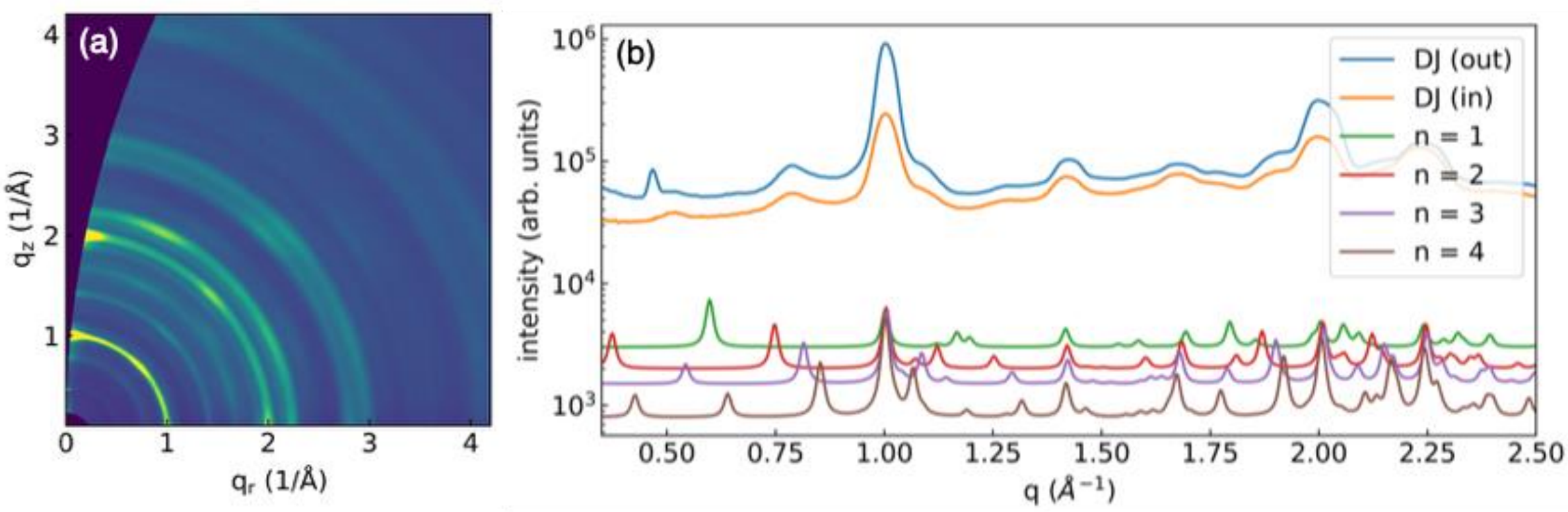

Figure S5. (a) 2D GIWAXS pattern and (b) corresponding integrated GIWAXS data along the out-of-plane and in-plane directions, with reflections assigned based on calculated diffraction patterns for DJ (4AMP) $(MA)_{n-1}Pb_nI_{3n+1}$ (n = 1, 2, 3, and 4) perovskites. The GIWAXS data was acquired at DELTA BL9 beamline using X-ray of 20 keV energy at an incident angle of 0.15°. Though a clear verification of mixed phases in 2D DJ perovskites is limited by resolution and probing depth of the measurement.

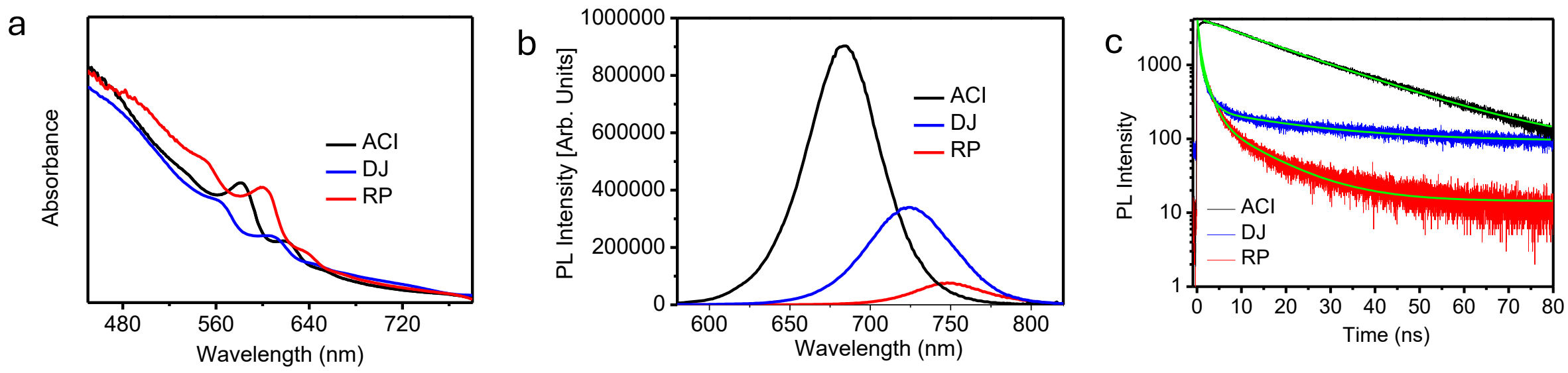

Figure S6. (a) Absorbance, (b) Steady state PL and (c) Time-resolved PL kinetics of higher order multiphase ACI3 (black), DJ4 (blue) and RP4 (red) perovskite. For PL excited at 400 nm and for PL kinetics excited at 400 nm and monitor at 684 nm, 726 nm and 750 nm.

Global and target analysis. Time-resolved spectra were analyzed globally using pyglotaran (v0.7.4).[10] Prior to fitting, singular value decomposition (SVD) of each $\Delta OD(t,\lambda)$ data matrix was performed to determine the number of significant kinetic components; a clear break in the singular-value spectrum indicated a minimum rank of three for ACI3 and four for RP and DJ (Figure S7), consistent with the greater structural complexity of the latter two systems. A sequential kinetic scheme ($S_1 \rightarrow S_2 \rightarrow \ldots \rightarrow S_n \rightarrow$ ground) convolved with a Gaussian instrument response function (IRF), including a third-order coherent-artifact term to account for pump–probe temporal overlap effects near $t = 0$, was fit to each dataset using variable projection, with linear species amplitudes solved analytically at each nonlinear iteration. Fits were validated by (i) confirming convergence to consistent parameter values and cost from multiple independent starting guesses, (ii) comparing reduced χ^2 across models with one fewer and one additional kinetic component relative to the SVD-indicated rank, and (iii) confirming that additional components beyond the validated rank produced statistically unconstrained, degenerate rate constants rather than genuine improvement.

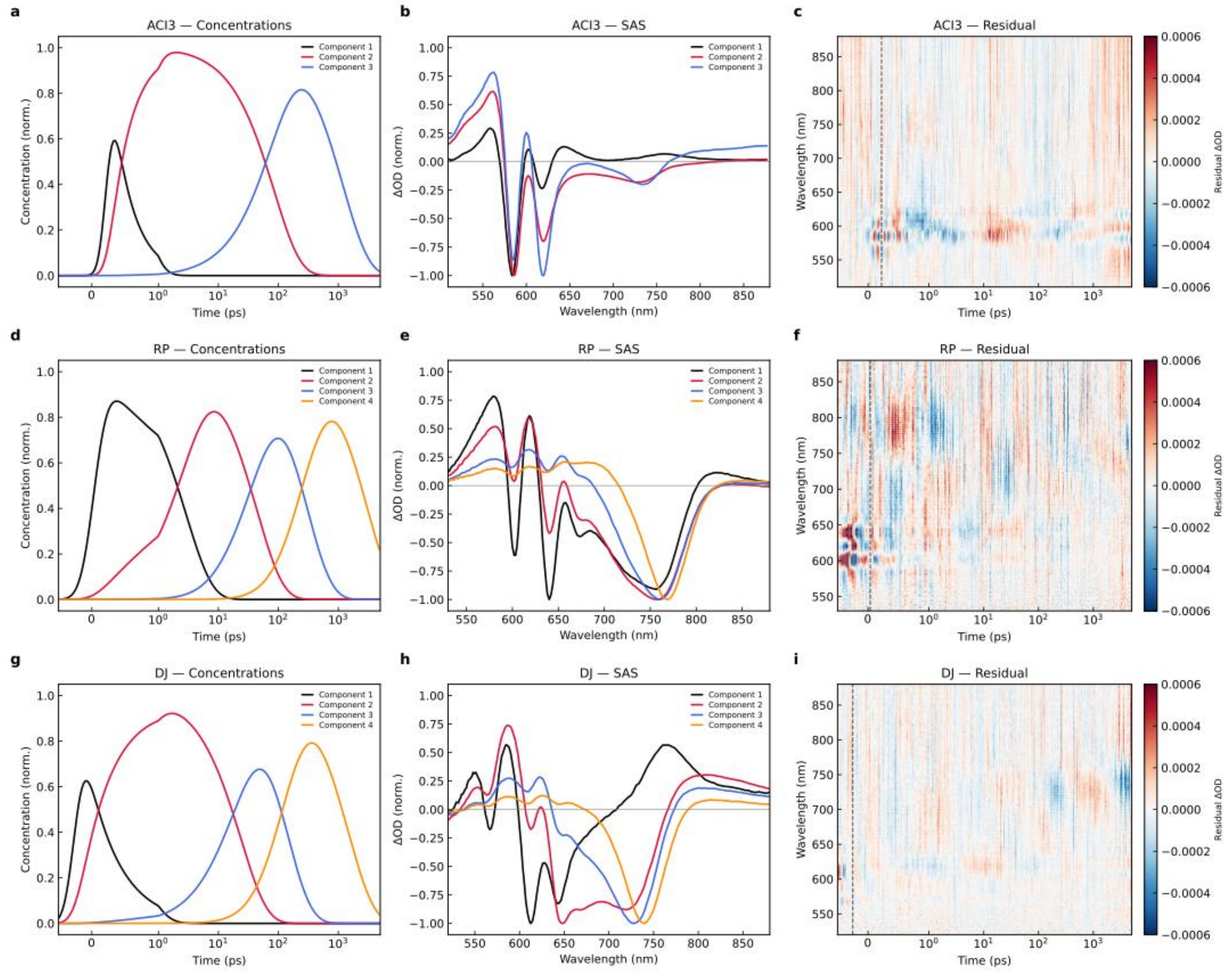

Figure S7. Global and target analysis of the transient absorption data for the ACI3, RP and DJ films. (a,d,g) Normalized concentration profiles of the three kinetic components obtained from sequential target analysis. (b,e,h) Corresponding normalized species-associated spectra (SAS). (c,f,i) Residual map of the global fit showing the difference between the experimental and fitted transient absorption data.

Table S2: Fitting parameters

Sample	τ_1 (ps)	τ_2 (ps)	τ_3 (ps)	τ_4 (ps)
ACI3	0.311 ± 0.025	85.6 ± 0.58	1190 ± 7	-
RP	2.9 ± 0.39	43.7 ± 0.58	277 ± 3	2960 ± 10

DJ	0.481± 0.033	23.7 ± 0.22	124 ± 1	1440 ± 5

References:

1. C. M. M. Soe, C. C. Stoumpos, M. Kepenekian, B. Traoré, H. Tsai, W. Nie, B. Wang, C. Katan, R. Seshadri and A. D. Mohite, *Journal of the American Chemical Society*, 2017, **139**, 16297–16309.
2. X. Li, W. Ke, B. Traoré, P. Guo, I. Hadar, M. Kepenekian, J. Even, C. Katan, C. C. Stoumpos and R. D. Schaller, *Journal of the American Chemical Society*, 2019, **141**, 12880–12890.
3. D. H. Cao, C. C. Stoumpos, O. K. Farha, J. T. Hupp and M. G. Kanatzidis, *Journal of the American Chemical Society*, 2015, **137**, 7843–7850.
4. X. Zhang, R. Munir, Z. Xu, Y. Liu, H. Tsai, W. Nie, J. Li, T. Niu, D. M. Smilgies and M. G. Kanatzidis, *Advanced Materials*, 2018, **30**, 1707166.
5. S. Ghosh, B. Pradhan, W. Lin, Y. Zhang, L. Leoncino, P. Chabera, K. Zheng, E. Solano, J. Hofkens and T. Pullerits, *The Journal of Physical Chemistry Letters*, 2023, **14**, 1066–1072.
6. H. Amenitsch, M. Rappolt, M. Kriechbaum, H. Mio, P. Laggner and S. Bernstorff, *Synchrotron Radiation*, 1998, **5**, 506–508.
7. M. Burian, C. Meisenbichler, D. Naumenko and H. Amenitsch, *Applied Crystallography*, 2022, **55**, 677–685.
8. Z. Jiang, *Applied Crystallography*, 2015, **48**, 917–926.
9. C. C. Stoumpos, D. H. Cao, D. J. Clark, J. Young, J. M. Rondinelli, J. I. Jang, J. T. Hupp and M. G. Kanatzidis, *Chem. Mater.*, 2016, **28**, 2852–2867.
10. I. H. van Stokkum, J. Weißenborn, S. Weigand and J. J. Snellenburg, *Photochemical & Photobiological Sciences*, 2023, **22**, 2413–2431.
